# Prickly Problems: *Cylindropuntia*'s Low Genetic Diversity Despite Inbreeding Avoidance

**DOI:** 10.1002/ece3.71213

**Published:** 2025-04-15

**Authors:** Niveditha Ramadoss, Scarlet Steele, Lluvia Flores‐Renteria

**Affiliations:** ^1^ Department of Biology San Diego State University San Diego California USA

**Keywords:** *Cylindropuntia*, dead‐end hypothesis, dioecy, genetic diversity, gynodioecy

## Abstract

Dioecy, the separation of sexes, is found in 6% of flowering plants. One widely known hypothesis suggests that it is an adaptation to mitigate inbreeding. A contrary hypothesis suggests that dioecy is an evolutionary dead end. However, contrasting patterns emerged from population genetic studies that compared the genetic diversity between dioecy versus hermaphroditic species. Specifically, in *Silene*, it was shown that dioecious species possess higher genetic diversity than hermaphroditic species, challenging the dead end hypothesis. To evaluate whether dioecy is indeed advantageous, further studies are needed in systems with diverse sexual systems such as the genus *Cylindropuntia* (Cactaceae). It encompasses species with sexual separation observed solely in polyploids. Notably, these polyploids ( *C. wolfii* and *C. chuckwallensis*) share similar ploidy, flower colors, and geographic proximity, raising speculation about their shared ancestry. Moreover, *C. wolfii* has been reported to have a low seed production, highlighting the need to assess the reproductive strategies of the species. Our first goal was to compare the genetic diversity patterns among species with different sexual systems within the genus *Cylindropuntia* (Cactaceae). Our second goal was to investigate genetic shared ancestry among the polyploid species. As *C. wolfii* is struggling to sexually reproduce, our third objective was to investigate whether it is dominated by clonal reproduction, diversity parameters, and population structure. The clonality of *C. wolfii* was assessed using a combination of field survey and genetic analysis. The pattern of genetic diversity in species with diverse sexual systems did not support the dead end hypothesis. The field survey of *C. wolfii* revealed no seed recruitment, but the genetic analysis on the current adult plants showed low signs of clonality, suggesting that this species has recently shifted to clonal reproduction. Results showed that overall, this genus had low genetic diversity and high differentiation, implying that it is vulnerable to environmental threats.

## Graphical abstract

Our research focuses on comparing the genetic diversity patterns among species with different sexual systems in the genus *Cylindropuntia*. We found that the *Cylindropuntia* genus does not support the dioecy dead end hypothesis because the species with bisexual flowers have a lower genetic diversity than that of sexually separated species.

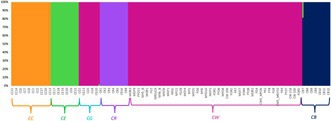

## 1 Introduction

The sexual systems found in angiosperms exhibit a wide range of diversity. Roughly 90% of flowering plants have a hermaphroditic system, containing both male and female reproductive structures within the same flower of an individual. Some plant species have developed floral unisexuality through spatial separation of their flowers (Yampolsky and Yampolsky 1922; Renner 2014). These plants may exhibit male and female reproductive structures on different flowers of the same plant, which is known as the monoecious sexual system or have male and female flowers on separate male and female plants, respectively, known as the dioecious sexual system (Darwin 1877; Barrett 2002). While the evolution of dioecy has been a source of fascination for plant biologists, it is infrequent, constituting approximately 6% of the total species in flowering plants (Renner 2014). The gynodioecy sexual system has been suggested to be one of the pathways for shifting from hermaphroditism to dioecy in angiosperms; thus, gynodioecious populations are composed of female individuals and hermaphroditic individuals (Darwin 1877; Renner 2014). Consequently, gynodioecy is often viewed as a less stable sexual system, characterized by its potential for reversibility (Charlesworth and Charlesworth 1978; Spigler and Ashman 2012).

Angiosperm species with individuals bearing hermaphroditic flowers can exhibit a mixed mating system, leading to seed production through both self‐pollination and cross‐pollination. Plant sexual systems, together with mating patterns, have played a key role in driving genome evolution (Charlesworth 1992; Charlesworth and Wright 2001; Haudry et al. 2008) and influencing genetic diversity levels in plant species (Charlesworth and Charlesworth 1995; Nazareno and de Carvalho 2009; Willi and Maattanen 2011; Muyle et al. 2021). There are two main hypotheses that can explain how genetic diversity is influenced by sexual systems and mating patterns.

One hypothesis is widely known and explains the separation of sexes in gynodioecious, and dioecious systems have evolved as an adaptive strategy to minimize the risks associated with inbreeding and the subsequent detrimental effects of inbreeding depression (Charlesworth and Charlesworth 1978; Olito and Connallon 2019). In gynodioecy, although females are obligate outcrossers, the presence of hermaphroditic individuals is associated with selfing and therefore potential inbreeding depression (Darwin 1877; Lloyd 1982; Charlesworth and Charlesworth 1978; Maki 1992). In contrast, dioecy achieves maximal outcrossing due to the absence of hermaphrodites in the population (Lloyd 1982; Charlesworth and Charlesworth 1978), thereby reducing the chances of inbreeding depression (Thomson and Brunet 1990; Charlesworth 1999). Under this hypothesis, without a genetic self‐incompatibility mechanism, the expectation is that dioecious species will have higher genetic diversity than gynodioecious which will be greater than that of hermaphroditic species (Muyle et al. 2021). Alternatively, the “dead‐end” hypothesis, as proposed by Heilbuth (2000), posits that dioecious lineages exhibit reduced species diversity in contrast to their non‐dioecious counterparts, possibly due to elevated rates of extinction. Under this hypothesis, the expectation is that dioecious species will have lower genetic diversity than that of hermaphroditic species (Heilbuth 2000; Muyle et al. 2021).

Only a few studies have compared genetic diversity between dioecious and hermaphroditic species, and these have revealed contrasting patterns (Maki 1992; Muyle et al. 2021). For example, in the dioecy model genus *Silene*, Muyle et al. (2021) discovered that both dioecious and gynodioecious species exhibited higher genetic diversity when compared to their nearby hermaphroditic counterparts. On the other hand, a similar study by Maki (1992) in the *Chionographis* genus showed that gynodioecious species have lower genetic diversity compared to their hermaphroditic close relatives. Even though there are studies estimating genetic diversity and inbreeding in species with different sexual systems (Nazareno et al. 2013), to the best of our knowledge, these contrasting studies (Maki 1992; Muyle et al. 2021) are the only two comparing the genetic diversity between species with different sexual systems in the same genus. Thus, further research is needed in systems with diverse sexual systems to assess whether dioecious species indeed have a higher genetic diversity than their hermaphroditic relatives. In our research, we focused on the genus *Cylindropuntia*, because it has hermaphroditic, gynodioecious and dioecious systems allowing us to evaluate Muyle et al. (2021)'s pattern of genetic diversity in contrasting sexual systems. Although the *Cylindropuntia* genus has diverse sexual systems, the dioecy/gynodioecy is only observed in polyploid species—*C. chuckwallensis*, *C. calmalliana* , *C. sanfelipensis* , *C. wolfii* and *C. molesta* (Baker and Cloud‐Hughes 2014; Ramadoss et al. 2022). Currently, there are no studies about the study species' sex‐determination system or its potential for self‐fertilization. Moreover, the coincidence of separate sexes, hexaploidy, diversity with up to geographic proximity and narrow distributions among *C. chuckwallensis*, and *C. wolfii* is remarkable (Baker and Cloud‐Hughes 2014). Baker and Cloud‐Hughes (2014) hypothesize that these two species may be of hybrid origin (allopolyploidy) and share ancestry but there is no genetic data to support this, and provide clear species delimitation for both. In response to the gap in knowledge regarding the shared ancestry of these two species, our study aimed to investigate this by population structure analysis.

The family Cactaceae belongs to the taxonomic groups facing the highest threat levels analyzed to date, with about 31% of the species classified in danger of extinction (Goettsch et al. 2015). The reported low seed set in *C. wolfii* , even with manual pollination (Ramadoss et al. 2022) has raised conservation concerns, prompting an understanding of the population's genetic variation. The low seed set suggests challenges in successful sexual reproduction (Ramadoss et al. 2022) of *C. wolfii* . This emphasizes the importance of investigating whether the *C. wolfii* population is predominantly reproducing through clonal ways. There is a lack of studies on other species of *Cylindropuntia* to assess if the seed set is similarly affected.

The Cactaceae family comprises approximately 2000 species (Anderson and Brown 2001), the majority of which are hermaphroditic. However, 23 species exhibit sexual separation in the form of dioecious, gynodioecious, or rarely trioecious sexual systems (Sánchez and Vázquez‐Santana 2018 and references therein). Within Cactaceae, the subfamily Opuntioideae is known to exhibit diversity in sexual systems (Wisnev 2024). For example, six *Consolea* taxa are reported to be dioecious: *C. microcarpa* , *C. millspaughii* , *C. moniliformis* , *C. nashii* , *C. rubescens* , and *C. spinosissima* (Strittmatter et al. 2008) while *C. spinosissima* is trioecious (Strittmatter et al. 2002). In the genus *Opuntia*, *O. stenopetala* was found to be functionally dioecious (Orozco‐Arroyo et al. 2012); *O. quimilo* is gynodioecious (Díaz and Cocucci 2003), and *O. robusta* consists of hermaphroditic, dioecious, and trioecious populations (Del Castillo and Argueta 2009). Five *Cylindropuntia* species were reported as gynodioecious: *C. calmalliana* , *C. chuckwallensis*, *C. molesta* , *C. sanfelipensis* , and *C. wolfii* (Wisnev 2024; Baker and Cloud‐Hughes 2014). Later, *C. wolfii* was identified to be functionally dioecious based on histological analysis and cross‐pollination experiments (Ramadoss et al. 2022). The sexual system of the subfamily Opuntioideae has been investigated at the ecological, morphological, and cellular level (Ramadoss et al. 2022, 2023; Flores‐Rentería et al. 2013; Strittmatter et al. 2008); however, it stands out for its limited number of genetic studies at the population level (Cariaga et al. 2005), marking a notable paucity in research (Nassar et al. 2002; Helsen et al. 2011; Guerrero et al. 2019). This genus encompasses about 39 species occurring throughout the major deserts of North America (Majure et al. 2019). Our study is the first to report genetic parameters for species of this genus. By exploring the genetic makeup of these species, we not only gain insights about sexual separation but also provide valuable data for conservation efforts. We included six species from this genus, each characterized by distinct sexual systems: three species featuring hermaphroditic sexual systems ( *C. echinocarpa* , *C. ganderi* , and *C. ramosissima* ), one gynodioecy (*C. chuckwallensis*), one dioecy ( *C. wolfii* ), and one hermaphroditic species with a clonal reproductive strategy ( *C. bigelovii* ).

Our first goal was to test whether the different sexual systems influence the genetic diversity in the species of *Cylindropuntia*. We expect higher genetic diversity in species with sexual separation compared to the hermaphrodites. Our second goal was to investigate whether there is shared ancestry between the two species *C. wolfii* and *C. chuckwallensis* that share similar morphological traits, including unisexual flowers, geographic proximity, and narrow distribution using clustering analysis. We expect that if the two species have shared ancestry, they will cluster closely or together. Our third objective was to investigate whether *C. wolfii* is dominated by clonal reproduction. We hypothesize that *C. wolfii* is clonally reproducing as it has been reported to have a reduced seed production (Ramadoss et al. 2022). This study lays a foundation for future research on dioecy evolution and conservation approaches concerning *Cylindropuntia*.

## 2 Materials and Methods

### 2.1 Sampling

To evaluate the genetic diversity pattern in the genus *Cylindropuntia*, we examined the variations in genetic diversity among three different sexual systems (hermaphroditism, gynodioecy, and dioecy) within the genus *Cylindropuntia*. For hermaphroditic sexual systems, we collected fragments of at least 15 individuals from four species— *C. echinocarpa* (CE), *C. ganderi* (CG), *C. bigelovii* (CB), and *C. ramosissima* (CR). For the dioecious sexual system, we collected 124 fragments of *C. wolfii* (CW), and for the gynodioecious system, we collected 30 fragments of *C. chuckwallensis* (CC) (Table S1). The species of CE, CG, CB, CC, and CR were collected from Joshua Tree National Park (JTNP), allowing a maximum of 30 samples, while CW was collected from BLM lands. All the species were collected from a single study site each. Our study is limited by the inclusion of only one dioecious and one gynodioecious species, as no other dioecious species are known within this genus, and no additional gynodioecious species are distributed in the United States.

All the hermaphroditic species we collected except *C. bigelovii* (triploid) are diploid, whereas the gynodioecious *C. chuckwallensis* and dioecious *C. wolfii* are hexaploid. There are no known gynodioecious nor dioecious diploid species in this genus. To mitigate any bias introduced by high ploidy levels within the system that is, higher diversity observed due to the hexaploid nature of the gynodioecious and dioecious species, we specifically collected specimens of a triploid species with a hermaphroditic reproductive system, known as *Cylindropuntia bigelovii* (CB). If the triploid species CB shows significantly higher genetic diversity than the diploids, it might indicate that the diversity observed in the *Cylindropuntia* species may be influenced by ploidy level, complicating direct comparisons. All the collected fragments were potted in the greenhouse and maintained for future analysis. We ensured to collect only diploid individuals of *C. ramosissima* by sampling from JTNP, CA, USA, as they are also found to have a tetraploid population in Arizona (Baker et al. 2009). Given the presence of diploid individuals of CB in the Chuckwalla Mountains (Baker and Pinkava 2018), we did not sample CB from that location. Preliminary observations suggest that the CB population we sampled in JTNP (from Cactus Garden Trail) reproduces vegetatively, which suggests our CB samples consist solely of triploids. We adopted a population genomics strategy, leveraging DArT‐seq technology, which has a complexity reduction step that effectively diploidizes polyploid data, generating thousands of SNPs from the same homeologs, useful for comparison (Sansaloni et al. 2020). The process by which SNPs are obtained from a single homeolog is by making use of sequence variations and methylation differences among homeologs. DArTseq clusters similar sequences and then separates them into SNP loci using specialized bioinformatics algorithms. During this process, paralogs are efficiently filtered out from the data (Sansaloni et al. 2020). Subsequently, these SNPs were subjected to analysis for the assessment of genetic diversity and population structure in each species. Moreover, DArtseq also efficiently targets low copy sequences using certain endonucleases to avoid repetitive DNA regions (Wenzl et al. 2004).

### 2.2 DNA Extraction and Sequencing

A small piece of tissue from the cholla fragments was collected and placed in silica beads for about 2 weeks. The tissue was carefully cut to remove just the epidermis and collenchyma part to avoid excess moisture. The nuclear DNA was extracted using a DNeasy Plant Mini kit by Qiagen. The DNA quality was assessed by running a 1% agarose gel electrophoresis. After confirming the presence of high molecular weight bands from gel electrophoresis, the samples were shipped to DArT for sequencing following Buck et al. (2020, 2023). This technology uses a fusion of complexity reduction techniques and next‐generation sequencing (NGS) platforms to genotype thousands of SNPs, as outlined by Jaccoud et al. (2001). Genome reduction is achieved by employing specific endonucleases that target regions of low‐copy DNA (Wenzl et al. 2004). Moreover, this technique has been known to be effective in genetic diversity studies of polyploids as well as non‐model organisms (Wenzl et al. 2004; Sohail et al. 2015; Shams et al. 2019). Despite the challenges of comparing genetic diversity parameters across species with varying ploidy levels, studies have leveraged DArTseq technology to address this issue, particularly in wheat varieties (Sansaloni et al. 2020; Sohail et al. 2015).

A total of 227 samples were sequenced, and a high‐density assay provided 47,857 SNPs. We further filtered the data for loci having < 100% reproducibility, call rates of ranges 100% (no missing data), 90%, 80%, 70%, and 60%, monomorphs, departures from Hardy–Weinberg equilibrium, and loci that have more than one locus per sequence tag. The filtered data underwent conversion into suitable file formats for subsequent genetic analyses through the utilization of the R program called dartR (Gruber et al. 2018).

### 2.3 Genetic Diversity

Different measures of genetic diversity and inbreeding were calculated using GenoDive (Meirmans and Van Tienderen 2004). The effective number of alleles (corrected for unequal sample size), expected heterozygosity (*H*_e_), observed heterozygosity (*H*_o_), and inbreeding coefficient (*F*_is_) were obtained for all the 227 individuals from the six species— *C. wolfii* , *C. chuckwallensis*, *C. echinocarpa* , *C. ganderi* , *C. ramosissima* , and *C. bigelovii* . Although *C. bigelovii* (CB) cannot successfully reproduce sexually, we calculated its diversity parameters under the theoretical assumption of random mating. This approach was taken because CB is the only species in our study that is both hermaphroditic and polyploid. The confidence intervals for the statistics were obtained by bootstrapping over loci (10,000 bootstraps). In our study, we conducted a one‐sample *t*‐test to compare the estimates of gynodioecious species with those of hermaphrodites, assuming that gynodioecious *C. chuckwallensis* represents the expected mean. We performed a similar analysis to compare the estimates of dioecious *C. wolfii* with those of hermaphrodites.

### 2.4 Genetic Differentiation and Population Structure Analysis

To analyze the shared ancestry of *C. wolfii* and *C. chuckwallensis*, we estimated genetic differentiation and population structure from 227 individuals from the six species— *C. bigelovii* (CB), *C. wolfii* (CW), *C. chuckwallensis* (CC), *C. echinocarpa* (CE), *C. ganderi* (CG), and *C. ramosissima* (CR). The *F*_st_ was obtained using the stamppFst command in R package *adegenet* using the Weir and Cockerham (1984) method. The 95% confidence interval and *p*‐values for significance were obtained after 1000 bootstraps. We utilized the 10,000 s of SNP loci obtained from different call rates (100%, 90%, 80%, 70%, and 60%) in four distinct complementary genetic clustering methods: This included non‐model‐based approaches such as Principal Coordinates Analysis (PCoA) and Discriminant Analysis of Principal Components (DAPC) and model‐based analyses like fastSTRUCTURE and ConStruct. The non‐model‐based approaches are quick methods to identify the number of distinct gene pools while the model‐based approaches give a quantitative description of an individual's membership to a gene pool (Jombart 2008; Bradburd et al. 2018). In case of strong isolation by distance, common model‐based clustering approaches like fastSTRUCTURE will tend to overestimate the number of discrete gene pools present. But ConStruct is able to isolate these effects (Bradburd et al. 2018). So, we carried out these different methods to cross‐validate our findings and get a more detailed understanding of genetic structure in our data. Different call rates were utilized to assess the consistency of results, like the approach outlined in Melville et al. (2017).

Principal Coordinates Analysis (PCoA), conducted using dartR (gl.pcoa.plot) (Gruber et al. 2019), used differences in allele frequencies to delineate the populations. Genetically unique clusters were ascertained through Discriminant Analysis of Principal Components (DAPC) in R, employing the *adegenet* package (Jombart 2008; Jombart et al. 2010). DAPC uses the Bayesian Information Criterion (BIC) for comparing different cluster solutions. The selection of the optimal cluster solution or model was based on the lowest BIC score, as the lowest score is given to the model that provides the best trade‐off between fit and complexity. Cross‐validation was performed using the xvalDapc command to determine the appropriate number of retained principal components (Jombart and Collins 2015). This command offers an optimization procedure in balancing the number of principal components (PCs) in the model. This involves using a training dataset with varying numbers of PCs to determine which number of PCs best predicts group membership in samples not included in the training data (Jombart and Collins 2015).

A Bayesian analysis of population clustering was executed using the software fastSTRUCTURE (Raj et al. 2014). Here, we employed the logistic prior with five cross‐validations on the dataset comprising 10,839 loci without any missing data. Subsequently, we raised the missing data threshold (call rates of 90%, 80%, 70%, and 60%) to incorporate additional SNPs, to observe if this inclusion provides a more detailed population structure. The selection of model complexity (*K*) was facilitated by the chooseK command in fastSTRUCTURE, which does model selection based on the model that best balances the fit and complexity for the data. Subsequently, the Q mean bar plots derived from the analysis were visualized using Microsoft Excel. If species clustered together, we performed a subsequent sub‐structuring analysis in fastSTRUCTURE for within‐cluster resolution. This involved utilizing the simple prior as we have well‐structured populations.

We also inferred the genetic structure patterns through an additional spatial analysis known as conStruct (Bradburd et al. 2018) with SNP call rates of 100%, 90%, and 60%. In spatial conStruct analyses, geographic data are utilized, and the assumption is that allele frequencies exhibit a positive covariance linked to geographic locations due to isolation by distance (Bradburd et al. 2018). We evaluated spatial models across *K* = 3 to *K* = 10 with 1 chain each. The performance of the MCMC runs were assessed by examining the trace plots of the posterior probabilities, for a “fuzzy caterpillar” appearance as suggested by Bradburd et al. (2018). The assessment of model performance involved a combination of cross‐validation with eight replicates and 1000 MCMC iterations and layer contribution scores as followed by Clark et al. (2022) and Pless et al. (2022). The determination of the optimal *K* value was therefore based on selecting the model that has the highest predictive accuracy and preserves discernible layer contributions.

### 2.5 Clonality Analysis

To test whether *C. wolfii* is dominated by clonal reproduction, we conducted clonality analysis in our SNP dataset. When employing nuclear markers to delineate multilocus genotypes, the potential for misallocation of individuals exists, attributed to scoring errors, PCR artifacts, and somatic mutations (Meirmans and Van Tienderen 2004). To mitigate this bias, we utilized the GenoDive program (Meirmans and Van Tienderen 2004), which groups individuals above certain threshold genetic differences (calculated based on an infinite allele model) into a clonal lineage. We used two replicates from a total of eight individuals each to account for scoring errors and somatic mutations that can contribute toward genetic distance. To establish a threshold for genetic differences among pairs of individuals while excluding scoring errors or differences arising from somatic mutations, we adopted the approach outlined by Wetzstein (2022). Here, the clonal threshold was determined by doubling the maximum observed error rate in the dataset—specifically, doubling the maximum genetic distance between replicated samples from the same individual.

Complementary to the genomic analysis, we conducted a field survey to estimate seedling recruitment of *C. wolfii* to confirm whether this species reproduces sexually. The study was conducted in Mountain Springs, focusing on a prominent area of *C. wolfii* . Four quadrants with a size of 20 × 20 m were selected. Manual scouting was done during daylight hours to thoroughly explore the designated area. We looked for juvenile plants that are not around an adult plant and that are < 10 cm tall to distinguish them from clonally propagated individuals. However, depending on the presence and abundance of dispersers, some sexually produced offspring may occur underneath the mother plant, making it difficult to spot. Therefore, we acknowledge that our estimate of the sexual reproduction rate could be underestimated. Observations were recorded manually for each quadrant, noting their location within the quadrants. The number of observations was manually counted to determine the prevalence of sexual reproduction in *C. wolfii*.

As CC has restricted distribution, we wanted to assess whether we sampled clonal individuals. So we performed a clonality analysis using the GenoDive software (Meirmans and Van Tienderen 2004) that groups individuals into clonal groups based on a genetic distance threshold, calculated according to the infinite allele model. The threshold was selected by identifying a value between two peaks in the frequency distribution of pairwise genetic distances (Tsujimoto et al. 2020). The first peak, representing small genetic distances, is assumed to result from somatic mutations or genotyping errors, while the second peak reflects greater variation between genotypes (Meirmans and Van Tienderen 2004).

## 3 Results

### 3.1 Genetic Diversity Analyses

**FIGURE 1:**
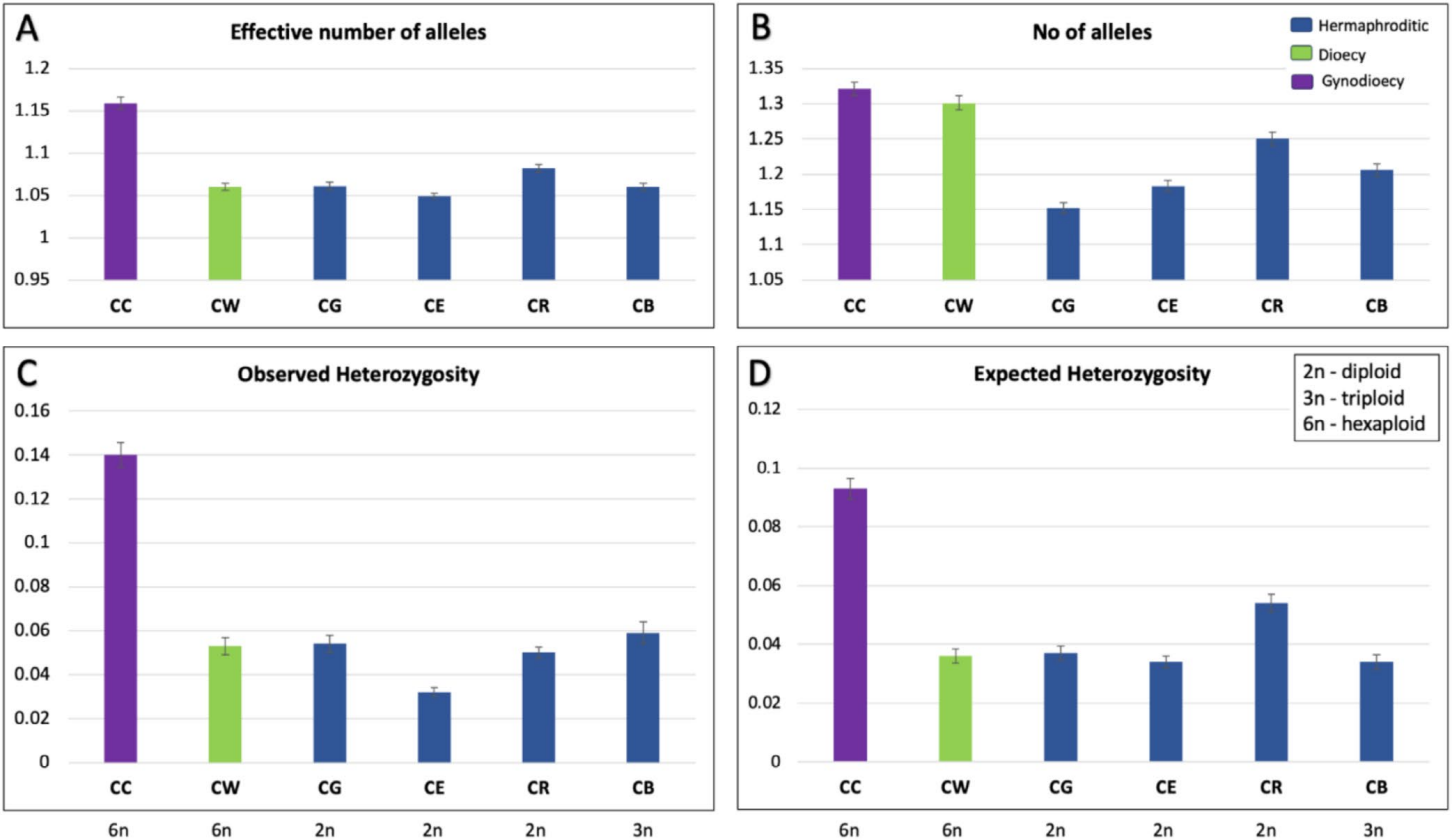
Genetic diversity parameters for gynodioecious (purple), dioecious (green), and hermaphroditic (blue) systems: (A) Effective number of alleles; (B) Number of alleles; (C) Observed heterozygosity; and (D) Expected heterozygosity. The error bars represent confidence intervals from bootstrapping (1000 resampling) over loci. *C. bigelovii* (CB), *C. chuckwallensis* (CC), *C. echinocarpa* (CE), *C. ganderi* (CG) and *C. ramosissima* (CR), and *C. wolfii* (CW). The bottom line indicates their corresponding ploidy.

**FIGURE 2:**
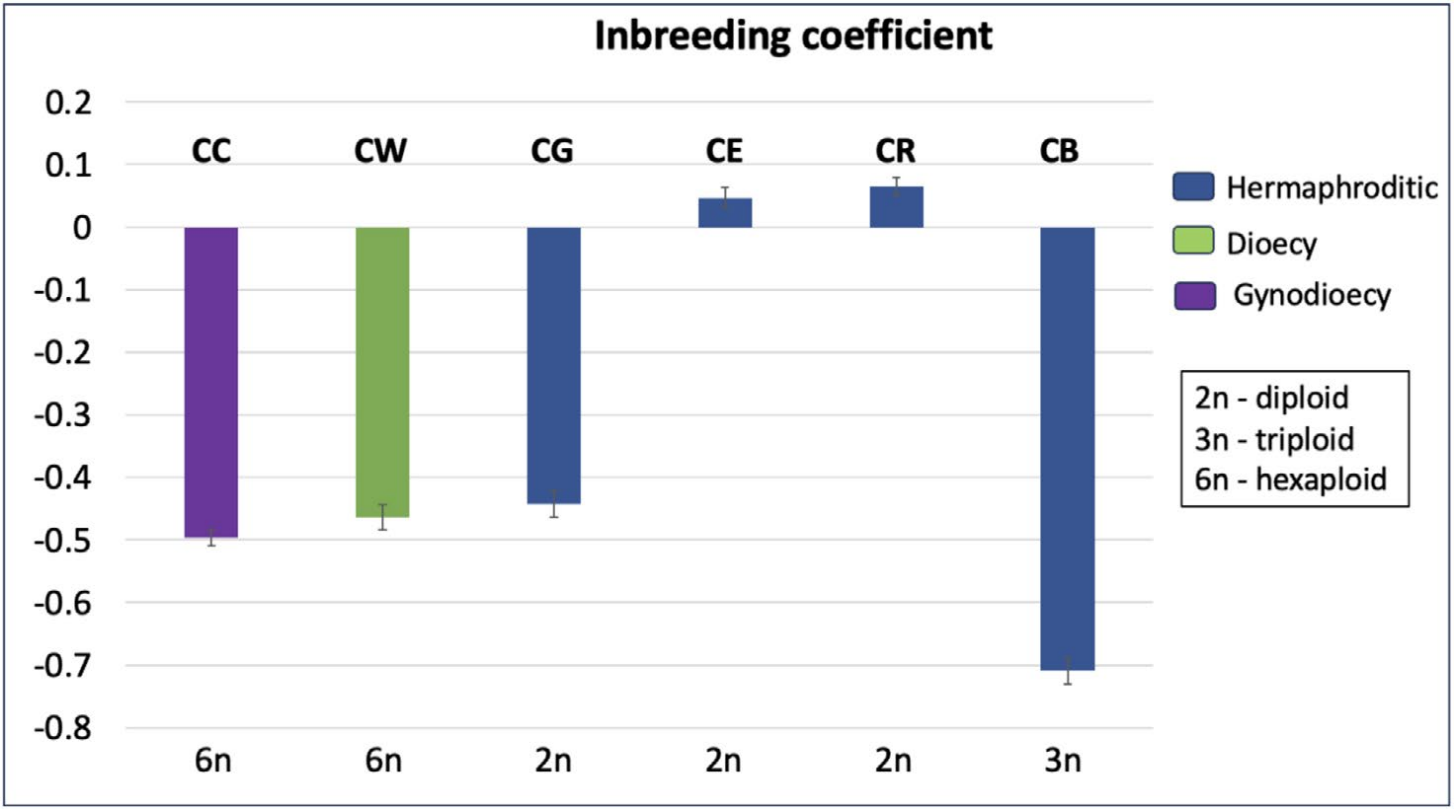
Inbreeding coefficient for gynodioecious (purple), dioecious (green), and hermaphroditic (blue) systems. The error bars represent confidence intervals from bootstrapping (1000 resampling) over loci. *Cistanthe bigelovii* (CB), *Cistanthe chuckwallensis* (CC), *Cistanthe echinocarpa* (CE), *Cistanthe ganderi* (CG) and *Cistanthe ramosissima* (CR), and *Cistanthe wolfii* (CW). The bottom line indicates their corresponding ploidy.

Following the implementation of the DArTseq method, a comprehensive set of SNP markers was identified. A 100% call rate yielded a total of 10,839 SNPs, followed by 23,563 SNPs for a 90% call rate, 33,157 SNPs for an 80% call rate, 39,190 SNPs for a 70% call rate, and 44,589 SNPs for a 60% call rate. To evaluate the genetic diversity pattern in the genus *Cylindropuntia*, we compared the genetic diversity between the sexually separated systems (gynodioecy and dioecy) and the hermaphroditic systems within the genus *Cylindropuntia*. The genetic diversity measures (Figure 1; Table S2) for every sampled population/species were estimated to be lower than those of their close relatives (Helsen et al. 2011). The observed heterozygosity (*H*_o_) estimates ranged from 0.032 to 0.14, with *C. chuckwallensis* having the highest. The expected heterozygosity (*H*_e_) estimates ranged from 0.034 to 0.093, with *C. chuckwallensis* again having the highest. In most cases, the observed heterozygotes were more than the expected values. For diversity in terms of effective number of alleles, *C. chuckwallensis* has the highest, followed by *C. ramosissima* . According to the *t*‐test results, it was found that *C. chuckwallensis* exhibited significantly higher values (*p* < 0.05) than its hermaphroditic counterparts across all genetic estimates except for inbreeding. Conversely, for *C. wolfii* , the *t*‐test indicated significantly higher values only in terms of the number of alleles (*p* = 0.02) when compared to its hermaphroditic counterparts. However, when we compare the effective number of alleles, which is corrected for unequal sample sizes, CC had a higher value than the hermaphrodites and CW had a comparable value. The estimated inbreeding coefficients for each population were low (Table S2; Figure 2), indicating avoidance or low levels of mating among closely related individuals in this genus.

### 3.2 Genetic Differentiation and Clustering Analyses

To assess hybrid origins or shared ancestry between CC and CW, we performed population differentiation and clustering analyses. *F*_st_ estimates have a value from 0 to 1, with lower numbers indicating lesser differentiation. Our results (Table 1) show high genetic differentiation between all species, except CW and CG, that are geographically closer. Estimated values range from 0.005 (between CG and CW) to 0.9 (between CE and CB). All the *F*_st_ values are significantly different from 0.

**TABLE 1:** Pairwise species *F*_st_ estimates between species.

Species	CC	CW	CG	CE	CR	CB
CC	0					
CW	0.4	0				
CG	0.295	0.006	0			
CE	0.452	0.629	0.634	0		
CR	0.433	0.683	0.641	0.735	0	
CB	0.798	0.881	0.883	0.9	0.873	0

*Note:* All values were significant. The shaded diagonal values are zero, representing comparisons of each species with itself.

Abbreviations: CB, *C. bigelovii* ; CC, *C. chuckwallensis*; CE, *C. echinocarpa* ; CG, *C. ganderi*; CR, *C. ramosissima* ; CW, *C. wolfii* .

**FIGURE 3:**
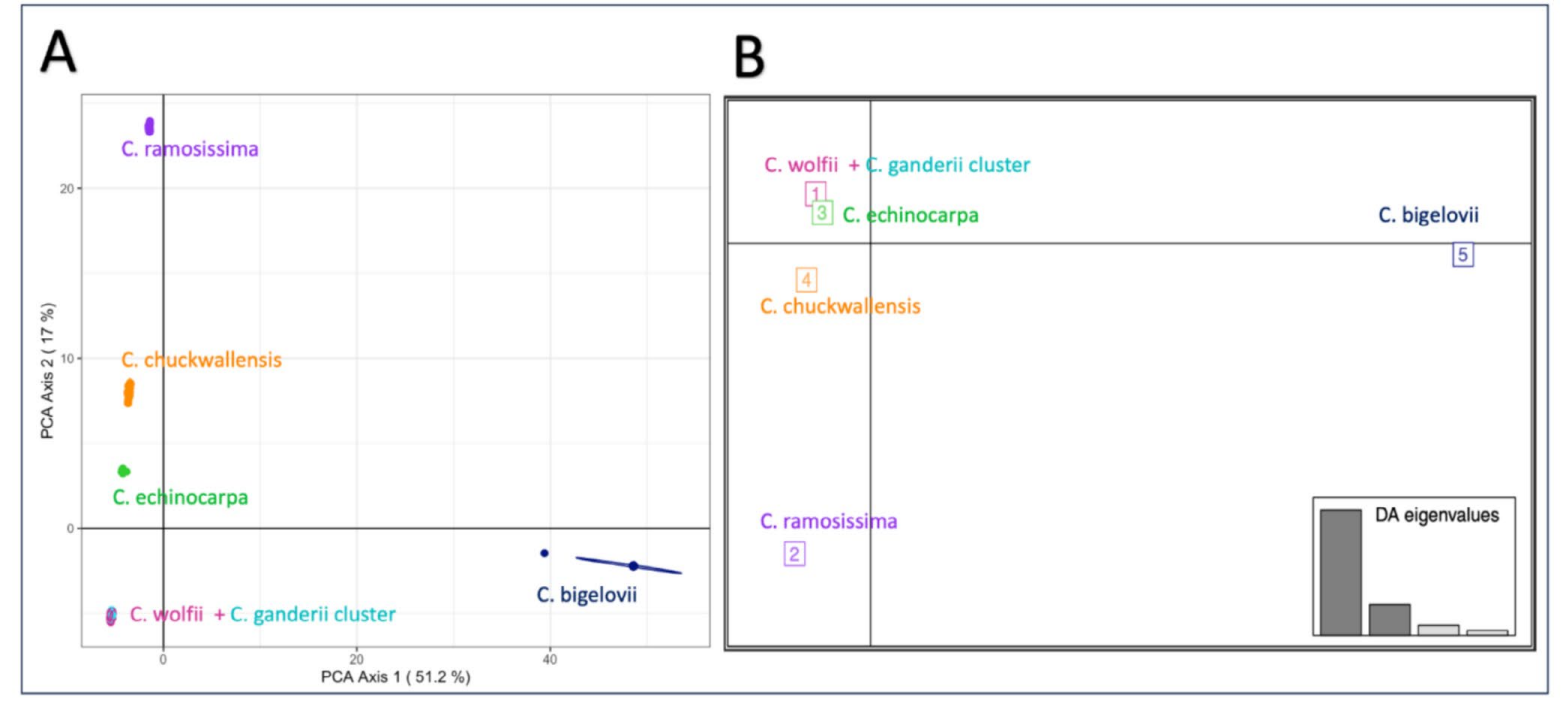
(A) Principal coordinate analysis (PCoA) of 227 samples using SNP markers with 100% call rate. The percentages of total variance explained by each coordinate are provided in parentheses. (B) DAPC for the same 227 samples and SNPs. The axes in this plot represent the first two linear discriminants (LD). Each square within the plot corresponds to a distinct cluster, and the numerical labels denote the different groups identified by DAPC.

The PCoA and DAPC results across all call rate thresholds were consistent and so, only the data from the 100% call rate are reported (Figure 3). PCoA indicated that the first two axes accounted for 68.2% of the variation, highlighting the separation of all 227 individuals into four distinct populations (Figure 3A). Notably, with the exception of individuals CG and CW, each group formed its own cluster based on its species group.

Consistent with PCoA, Discriminant Analysis of Principal Components (DAPC) also showed the formation of five genetically distinct clusters corresponding to the species (Figure 3B). Intriguingly, individuals from CG and CW were observed to share the same cluster. Then we further did hierarchical analysis of PCoA and DAPC including just CG, CW, CC, and CE and recovered three different groups that account for CG–CW cluster, CC, and CE respectively (Figure S1). Subsequent sub‐DAPC and sub‐PCoA analysis focusing just on CG and CW reaffirmed their joint clustering. In revised analyses of complete and subgroups, where the call rates were adjusted to 90%, 80%, 70%, and 60%, resulting in an increased number of SNPs, the genetic data did not show a clear separation between CG and CW.

**FIGURE 4:**
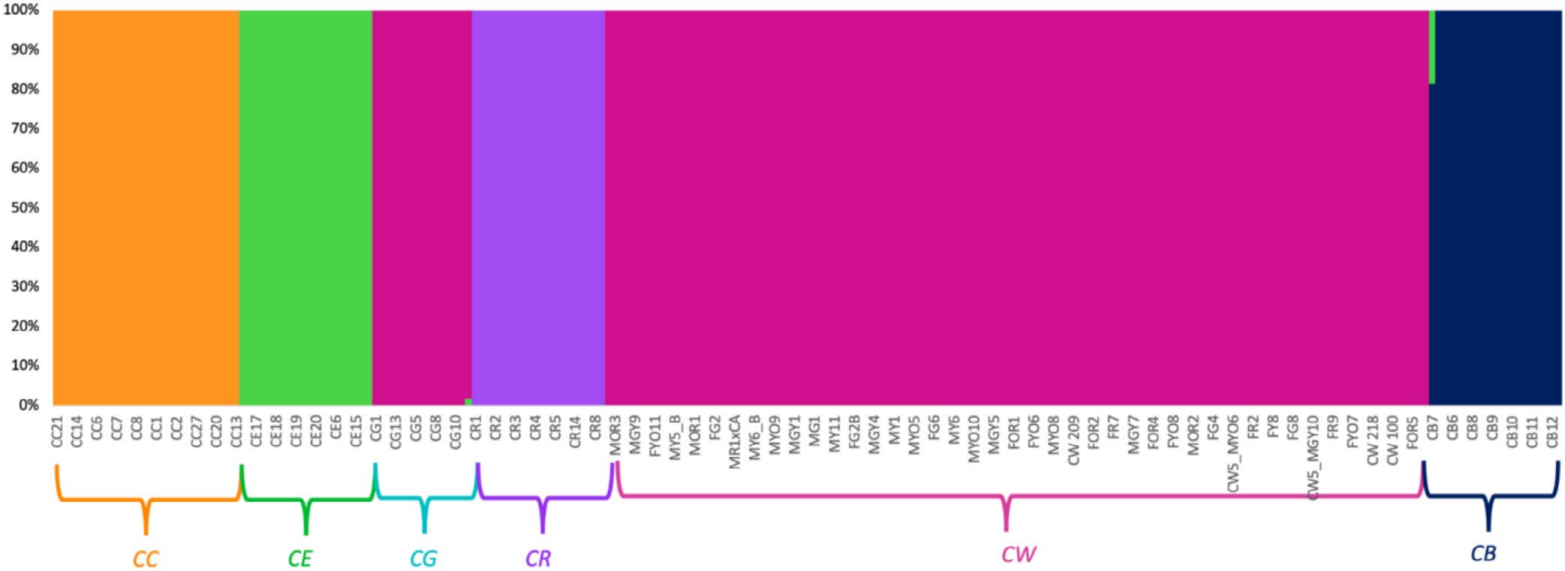
fastStructure plot showing five genetic clusters (*K* = 5) colored by genetic identity (orange = *C. chuckwallensis*, green = *C. echinocarpa* , purple = *C. ramosissima* , pink = *C. wolfii* + *C. ganderi* , and navy = *C. bigelovii* ) for 100% call rate. Each line on the *x*‐axis represents an individual and the proportion of ancestry derived from a certain genetic cluster is represented by the *y*‐axis. The species abbreviations are as follows: *C. bigelovii* (CB), *C. chuckwallensis* (CC), *C. echinocarpa* (CE), *C. ganderi* (CG), *C. ramosissima* (CR), and *C. wolfii* (CW).

FastStructure analysis showed that *K* = 5 to be the optimal number of clusters across all call rates. Again, CW and CG formed one cluster, and the rest of them formed their own cluster based on the species they belong to. No signs of admixture were evident from the plot (Figure 4). Substructuring using just CW, CG, CC, and CE with call rates ranging from 100% to 60% showed that *K* = 3 is the optimal number of clusters. Here again, CW‐CG was one cluster, and the other two species formed their own cluster (Figure S2). Further substructuring with CW and CG samples only and with 100%, 90%, 80%, 70%, and 60% missing data showed that *K* = 1 to be the optimal number of clusters.

**FIGURE 5:**
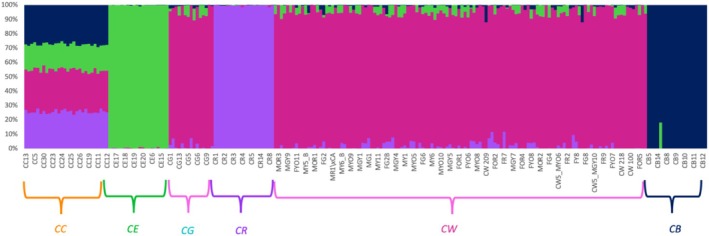
Construct plot based on 0% missing data, showing four genetic clusters (*K* = 4) colored by genetic identity (green = *Cylindropuntia echinocarpa* , purple = *Cylindropuntia ramosissima* , pink = *Cylindropuntia wolfii* + *Cylindropuntia ganderi* , and navy = *Cylindropuntia bigelovii* ). Each line on the *x*‐axis represents an individual and the proportion of ancestry derived from a certain genetic cluster is represented by the *y*‐axis. The species abbreviations are as follows: *C. bigelovii* (CB), *C. chuckwallensis* (CC), *C. echinocarpa* (CE), *C. ganderi* (CG) *C. ramosissima* (CR), and *C. wolfii* (CW).

The CONSTRUCT analysis, based on 0% missing data, indicated that *K* = 4 is the optimal number of clusters, as determined by layer contribution analysis and cross‐validation analysis (Figure 5). The four clusters were separated as follows: CE, CR, CB, and CW‐CG, and CC was observed to have shared ancestry from all the four clusters mentioned earlier, with genes derived almost equally from all four distinct clusters. However, in the presence of 10% missing data and an increased number of SNPs, the analysis suggested that *K* = 5 is optimal based on layer contribution and cross‐validation analysis (Figure S3). The same results were obtained for 40% missing data. This outcome aligns closely with the results obtained from FastStructure analysis. Once again, CW and CG clustered together, forming one distinct group, while the remaining entities constituted a separate cluster based on their respective species. Notably, in spatial analysis, indications of admixture were present, particularly in CC, CR, CW, and CG. In CC, there was discernible ancestry derived from CR, CE, CB, and CW (Figure S3).

### 3.3 Clonality Analysis and Field Survey

Our results showed partial evidence of clonal reproduction in *C. wolfii* . The maximum clonal distance (i.e., pairwise individual genetic distance which is the number of mutation steps needed to convert genotype of individual 1 to individual 2; Meirmans and Van Tienderen 2004) between the replicated samples was computed to be 78. Doubling this value results in a threshold of 156, and this was employed in the analysis to identify different genets. Out of the 124 individual *C. wolfii* ramets subjected to genotyping, GenoDive identified a total of 112 distinct clonal lineages plus six clonal groups with two ramets each.

In contrast, no new recruitment of *C. wolfii* seedlings was detected in the field as evidence of sexual reproduction. Out of the four surveyed quadrants, one quadrant revealed the presence of a juvenile cactus (32°40′27″ N, 116°05′51.0″ W) exhibiting characteristics suggestive of seed germination. However, it is highly probable that this juvenile cactus belongs to the species *Echinocereus engelmannii* , as it is native to the area. Notably, we did not encounter any seedlings of *Cylindropuntia* spp. during our investigation. Contrastingly, an average of 39 fragments per quadrant was observed in proximity to adult plants within the surveyed area.

We also found evidence of clonal reproduction in *C. chuckwallensis*. To distinguish different genets, we applied an objective threshold of 551, based on the midpoint between the two peaks in the bimodal histogram of genetic distance and the number of ramet pairs (Douhovnikoff and Dodd 2003). Among the 28 *C. chuckwallensis* ramets genotyped, GenoDive identified 18 distinct clonal lineages and four clonal groups. Each clonal group comprised two ramets, except for Group 2, which included eight ramets.

## 4 Discussion

The genetic diversity comparisons between species revealed that the genus *Cylindropuntia* did not follow the same genetic diversity pattern as that of *Silene* (Muyle et al. 2021) where genetic diversity of dioecious species > gynodioecious > hermaphrodites. Here, gynodioecious species CC exhibited significantly higher genetic diversity compared to all hermaphroditic counterparts. Interestingly, dioecious species CW demonstrated comparable genetic diversity to that of hermaphrodites, except in the number of alleles parameter. But this observation of the high number of alleles in CW was influenced by the larger number of samples compared to other species in our dataset which has been corrected in “effective number of alleles.” According to Muyle et al.'s (2021) study, species with separate sexes exhibited higher genetic diversity compared to hermaphrodites, as they rely on outcrossing, with the extent depending on the specific sexual system. Alternatively, another study comparing genetic structure between dioecious and monoecious species found that monoecious species exhibited weaker genetic structure than dioecious species (Nazareno et al. 2013). This difference was attributed to the long‐distance pollen dispersal mechanism in monoecious *Ficus* species indicated by their high genetic diversity (Nazareno et al. 2013). Although genetic diversity was not directly measured in dioecious *Ficus* species, the researchers speculated that it might be lower due to their higher genetic differentiation (Nazareno et al. 2013). Based on data from other studies, Nazareno et al. (2013) observed instances of inbreeding in several dioecious *Ficus*, while inbreeding was absent in the monoecious species studied. Their findings highlight that the long‐distance pollen dispersal mechanisms in monoecious *Ficus* play a crucial role in maintaining high genetic diversity and preventing inbreeding. In contrast, dioecious species typically exhibit lower pollen dispersal distances, contributing to reduced genetic diversity and a higher likelihood of inbreeding. Their theory aligns with Heibuth's theory, which suggests that dioecious species have reduced seed dispersal compared to cosexual species. However, our findings do not fully align with any of these expectations, although additional replication within *Cylindropuntia* species is necessary before drawing definitive conclusions. The low genetic diversity observed in CW may be linked to its reduced seed set (Ramadoss et al. 2022), potentially due to underlying genetic factors. It is also important to acknowledge that other factors, such as geographic distribution, gene flow, or population size, may contribute to the observed patterns of genetic diversity, independent of the sexual system. Although Copy Number Variations (CNVs) can also contribute to increased observed heterozygosity than expected, we believe this is unlikely in our case, as we utilized DArTseq technology, which effectively captures SNPs from low‐copy genomic regions (Wenzl et al. 2004). The inbreeding coefficients reveal that both the gynodioecious and dioecious systems exhibit outbreeding while some hermaphroditic species (CE and CR) have slightly positive Fis values which is suggestive of minor inbreeding. These findings would provide further support for the inbreeding avoidance hypothesis, suggesting that sexual separation in *Cylindropuntia* species may indeed have evolved to avoid inbreeding. Interestingly, the hermaphroditic CG displayed negative Fis values indicative of outbreeding (Weir and Cockerham 1984) as reported in other systems such as *Zostera marina* (Medina et al. 2024) and *Cypripedium macranthos* (Wu et al. 2023), despite the presence of bisexual flowers, suggesting the potential presence of self‐incompatibility strategies. The potential outbreeding in CC and CW can be attributed to the inability of females in the gynodioecious population and that of all individuals in dioecious populations to self‐fertilize. *Cylindropuntia chuckwallensis* is morphologically classified as gynodioecious; however, experimental crosses or embryological studies are needed to accurately determine the sexual system of this species (Ramadoss 2022). Therefore, it is possible that CC is functionally dioecious as CW. In such case, CC would rely entirely on outcrossing, explaining the observed high levels of outbreeding. Furthermore, CC is also hypothesized to be of hybrid origin from *C. acanthocarpa* and *C. multigeniculata* (Baker and Cloud‐Hughes 2014) based on morphological similarities (tepal and stigma color from *C. acanthocarpa* and stem number per trunk, habit from *C. multigeniculata*) and could have led to high levels of heterozygosity if the samples we collected were F1 hybrids (Buck et al. 2020). Hybridization is a frequent occurrence among chollas and can result in the formation of fertile allopolyploid species (Pinkava 2002; Mayer and Rebman 2021). Although we did not include *C. acanthocarpa* and *C. multigeniculata*, the putative parental species hypothesized by Baker and Cloud‐Hughes (2014), our clustering analysis indicates that CC likely originated from hybridization. The CB species is postulated to engage in clonal reproduction, a characteristic attributed to its triploid nature (Fong et al. 2019). However, the elevated outbreeding coefficient for CB observed in our study suggests an alternative explanation. This phenomenon can be explained by the Meselson effect, a mechanism known to sustain or potentially enhance heterozygosity over successive generations in asexual species through irreversible accumulation of mutations in homologous chromosomes (Mark Welch and Meselson 2000).

Overall, the genetic diversity values of *Cylindropuntia* were extremely low and comparatively similar to that of the critically imperiled Florida Tree cactus *Pilosocereus robinii* measured from SNP markers, also reported to have lower genetic diversity (Fotinos 2013). The inbreeding coefficient observed in *Cylindropuntia* is comparatively lower than that reported for *P. robinii* , a species where inbreeding is known to impact genetic diversity (Fotinos 2013). Hence, this implies that factors besides inbreeding may be influencing the reduced heterozygosity levels in *Cylindropuntia*. We can attribute it to genetic drift as it tends to have a more pronounced effect in small populations (Nabutanyi and Wittmann 2022). Additionally, in dioecious species with insect pollination, competition among males could render them more appealing to pollinators than females, thereby causing the overall population size to be contingent upon variations in pollinator density (Vamosi and Otto 2002). Consequently, fluctuating population sizes will lead to low levels of genetic diversity (Muyle et al. 2021). A previous study on *C. wolfii* demonstrated a pollinator preference bias toward males over female flowers (Ramadoss et al. 2023), attributed to their larger flower size and more vibrant colors. Thus, this phenomenon might elucidate the observed low genetic diversity in *C. wolfii* in our current study.

Genetic differentiation and clustering analyses not only help us understand the ancestry of species but also provide parameters for effective biodiversity conservation (Pearse and Crandall 2004; Ottewell et al. 2016; Buck et al. 2023). According to the qualitative indices for *F*_st_, the findings in *Cylindropuntia* species suggest high genetic differentiation, with the exception of CG and CW, implying strong differentiation among five out of six species of *Cylindropuntia* included in this study. Based on morphological characteristics such as prominent spines on upper stems, hiding stems, and tubercles (Chester 2007), it can be discerned that CW is more closely related to the CG clade than to the CE or CR clades (Majure et al. 2019). This finding aligns with the recent topology of *Cylindropuntia* constructed by Mayer and Rebman (2021), where CW is found to be more closely related to CG than it is to CR or CE. Additionally, our genetic clustering analysis like PCoA, DAPC, ConStruct, and FastSTRUCTURE also supplements the data by suggesting that there are five genetically distinct clusters grouping according to their species, except for the CW‐CG cluster. The clustering of CW and CG could be attributed to several plausible explanations. Firstly, it is possible that ongoing hybridization events are occurring between CG and CW. Secondly, considering CW is hypothesized to have a hybrid origin, it is plausible to speculate that CG may serve as one of its parent species. Additionally, given that Dartseq diploidizes the polyploid data, it is conceivable that the captured SNPs may represent the haplotype of one parent, potentially CG in this context. Alternatively, the genetic markers we sequenced may not have completely sorted into distinct lineages as not enough time has passed (ILS—Incomplete Lineage Sorting). Since they grow in sympatry, there is also a chance of convergence to similar genetic variations. Additional data such as Whole Genome Sequencing or conducting more advanced coalescent‐based phylogenetic analyses may help elucidate the underlying processes contributing to the clustering of CW‐CG.

Notably, the next pair with the least genetic differentiation are the two hexaploids CC and CW, which share similar flower color polymorphism, unisexual flowers, and a predicted shared common ancestor of *C. acanthocarpa* (Baker and Cloud‐Hughes 2014). In the DAPC and PCoA plots, CC clustered close to the CW and CG cluster, suggesting it could be phylogenetically closely related to them. Although no shared ancestry between CC and CW was evident in the FastSTRUCTURE analysis, we observed admixture between CC and CW individuals in the ConStruct analysis. Specifically, in both the *K* = 4 model, the CC cluster consisted of equally shared ancestry with all the other four gene clusters, while the CW individuals exhibited some admixture with the other four gene pools, implying the potential for an allopolyploid origin. In the *K* = 5 model, CC exhibited its own gene pool with admixture with all the other four gene clusters. This implies that the other species may either be the parent or share genetic material with the parents of CC. Furthermore, the low genetic diversity and the evidence for clonal reproduction in CC explain the consistent proportions of genetic ancestry from the various gene clusters observed across all CC individuals in the ConStruct analysis. Comprehensive phylogenetic studies including CC and CW will help shed more light on their evolutionary relationships and divergence. In the phylogenetic context, CB is recognized as the sister taxon to the remaining members of the *Cylindropuntia* genus, according to the study by Majure et al. 2019 and Mayer and Rebman (2021), thereby accounting for its high genetic differentiation from other species within the group in our dataset.

The overall low genetic diversity raises significant conservation concerns for the *Cylindropuntia* species (Fahrig 2002; Reed and Frankham 2003). The low genetic diversity is directly related to a constrained evolutionary potential (Spielman et al. 2004). Consequently, the six *Cylindropuntia* species in our study might encounter challenges in adapting to forthcoming extreme climatic changes (Zscheischler et al. 2020). Some species have overcome this low genetic diversity with the help of gene flow (Teukeng et al. 2022; Buck et al. 2023). But our data do not show evidence for a lot of gene flow in these species except for CC, potentially because of differences in ploidy or other kinds of barriers that could be temporal, mechanical, or behavioral.

The observed clonality from genetic markers does not account for the low genetic diversity within the *C. wolfii* population. Excluding the known replicates, two clonal lineages were identified, each comprising a pair of ramets from different individuals. In one lineage, the individuals were geographically proximate (50 ft), both being females. However, one had orange‐red flowers, while the other exhibited a red flower morph. This shows that even though they are from a single clonal group, they can accumulate genetic variations that can influence a slight shift in their flower color morphs or that flower colors exhibit some phenotypic plasticity. The second clonal pair, despite being 365 ft apart, consisted of both male individuals with green flower color morphs. This suggests limited clonal recruitment in the adult population/current generation that exists in Mountain Springs. However, during our sampling process, we specifically collected individuals that were at least 20 ft apart. Thus, it is possible that our sampling method may have led to an underestimation of clonality among the adult plants present in Mountain Springs. The observations from the field survey, however, suggest a phenomenon consistent with clonal propagation for the next generation, as the fragments we noted are likely a result of asexual reproduction process associated with falling of fragments from mature plants. Thus, one hypothesis is that this species has shifted from sexual reproduction to clonal reproduction, and this shift could be influenced by climate change, decrease in pollinators, negative mutation, or a combination of all such factors (Barrett et al. 2009; Barrett 2015; Hamann et al. 2021). Alternatively, sexual reproduction might be occurring in certain years under specific environmental conditions, and the year of our field survey may not have coincided with these conditions.

## 5 Conclusion

Based on the findings of our study, it is evident that the *Cylindropuntia* system does not follow the same genetic diversity pattern as expected by the dead‐end hypothesis. The dioecious system displayed genetic diversity parameters that were comparable to those of hermaphrodites. Interestingly, the gynodioecious species exhibited higher genetic diversity compared to the hermaphrodite species, providing partial support for the inbreeding avoidance hypothesis. However, it is crucial to acknowledge the ambiguity about the sexual system of *C. chuckwallensis*, as it is only morphologically identified as gynodioecious. Nevertheless, it is imperative to recognize the limitations of our study. The inclusion of only one dioecious and one gynodioecious species restricts the generalizability of our conclusions, as observed patterns may be influenced by factors beyond the sexual system itself. Furthermore, our genetic structure analysis revealed intriguing clustering patterns with CW‐CG forming one cluster, suggesting potential hybridization or incomplete lineage sorting, with *C. ganderi* possibly serving as one of the parents of *C. wolfii* . Overall, our investigation highlights the relatively low genetic diversity within the *Cylindropuntia* genus, with *C. wolfii* exhibiting a notable shift toward clonal reproduction, as indicated by field surveys. These findings underscore the urgent need for conservation efforts to protect these species and their genetic diversity.

## Author Contributions

**Niveditha Ramadoss:** conceptualization (supporting), formal analysis (lead), funding acquisition (supporting), investigation (supporting), methodology (lead), writing – original draft (lead). **Scarlet Steele:** investigation (supporting), methodology (supporting), writing – review and editing (supporting). **Lluvia Flores‐Renteria:** conceptualization (lead), funding acquisition (lead), investigation (supporting), methodology (supporting), project administration (lead), resources (lead), supervision (lead), validation (equal), visualization (equal), writing – original draft (lead), writing – review and editing (equal).

## Conflicts of Interest

The authors declare no conflicts of interest.

## Supporting information


Appendix S1

## Data Availability

SNP Reports are submitted in DRYAD with DOI: https://doi.org/10.5061/dryad.qbzkh18v7.
