# Supplementary material for: Prickly Problems: Cylindropuntia's Low Genetic Diversity Despite Inbreeding Avoidance
Source: Ecol Evol. 2025 Apr 15;15(4):e71213. doi: 10.1002/ece3.71213 (PMC11997463; doi:10.1002/ece3.71213)
Supplement: Supplementary file 1 — Appendix S1 [file ECE3-15-e71213-s001.docx]

**Supplementary Table 1.** Sampling locations with coordinates and number of individuals sampled per population.

| **Species** | **Lat** | **Long** | **Location** | **Individuals** |
| --- | --- | --- | --- | --- |
| *C wolfii* | 32.672 | -116.097 | Mountain Springs, Imperial county, CA | 124 |
| *C ganderii* | 32.677 | -116.104 | Mountain Springs, Imperial county, CA | 15 |
| *C chuckwallensis* | 33.733 | -115.812 | Joshua Tree National Park, CA | 28 |
| *C ramosissima* | 33.733 | -115.812 | Joshua Tree National Park, CA | 20 |
| *C echinocarpa* | 33.792 | -115.792 | Joshua Tree National Park, CA | 20 |
| *C bigelovii* | 33.925 | -115.928 | Joshua Tree National Park, CA | 20 |

**Supplementary Table 2: Genetic diversity estimates for each species. The species abbreviations are as follows:** *C. bigelovii* (CB)*, C. chuckwallensis* (CC), *C. echinocarpa* (CE)*, C. ganderii* (CG) *and C. ramosissima* (CR), *C. wolfii* (CW).

| **Species** | **No of samples** | **No of alleles** | **Effective number of alleles** | **Ho** | **He** | **Inbreeding coefficient** | **Sexual system** |
| --- | --- | --- | --- | --- | --- | --- | --- |
| CC | 28 | 1.321 | 1.159 | 0.14 | 0.093 | -0.497 | Gynodioecious |
| CW | 124 | 1.301 | 1.06 | 0.053 | 0.036 | -0.464 | Dioecious |
| CG | 15 | 1.152 | 1.061 | 0.054 | 0.037 | -0.443 | Hermaphroditic |
| CE | 20 | 1.183 | 1.049 | 0.032 | 0.034 | 0.047 | Hermaphroditic |
| CR | 20 | 1.25 | 1.082 | 0.05 | 0.054 | 0.065 | Hermaphroditic |
| CB | 20 | 1.206 | 1.06 | 0.059 | 0.034 | -0.709 | Hermaphroditic |


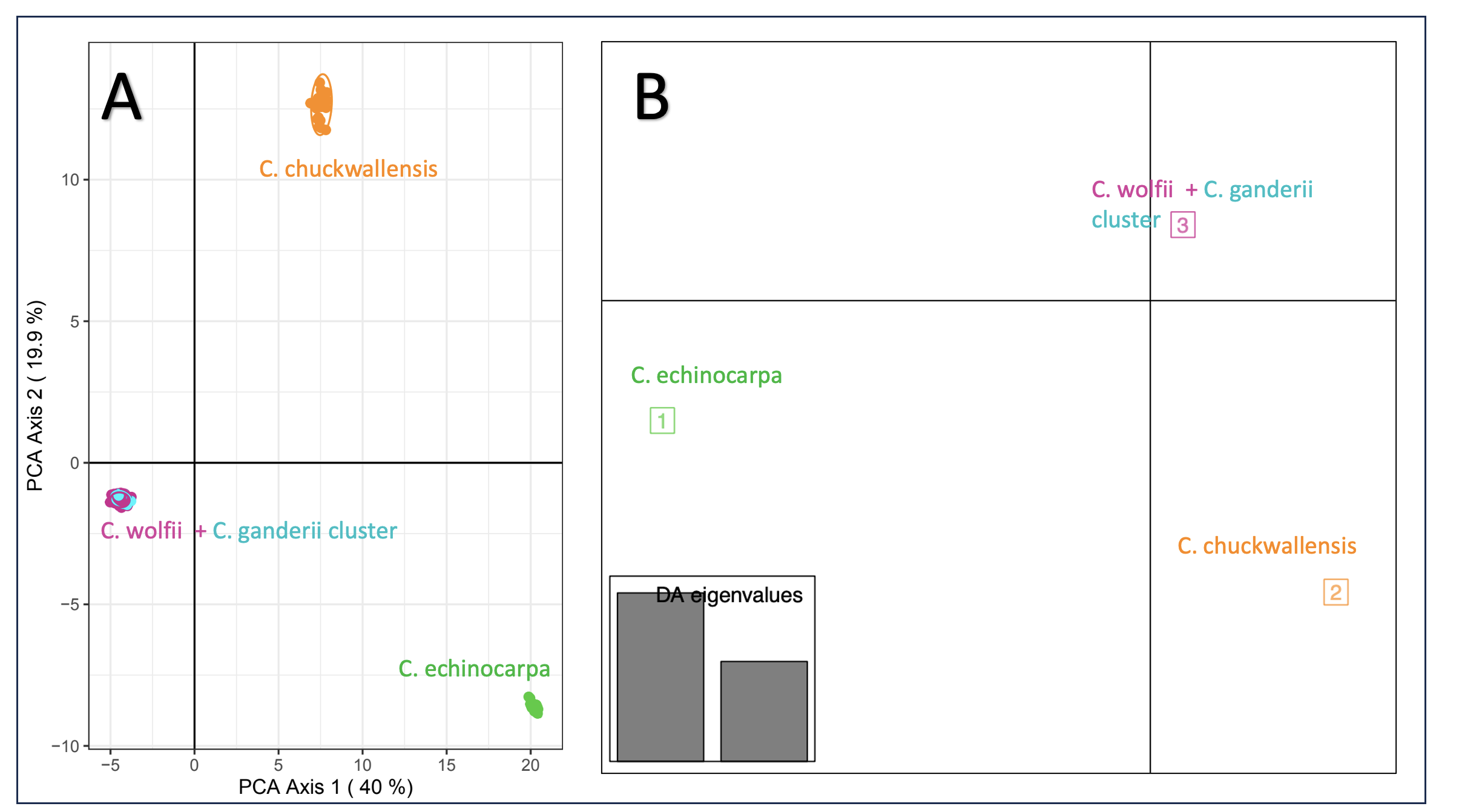


**Supplementary Figure 1. (A)** Principal Coordinate Analysis (PCoA) of CW,CG,CC and CE samples using SNP markers. The percentages of total variance explained by each coordinate are provided in parentheses. **(B)** Discriminant Analysis of Principal Components (DAPC) for the same CW,CG,CC and CE samples. The axes in this plot represent the first two linear discriminants (LD). Each square within the plot corresponds to a distinct cluster, and the numerical labels denote the different groups identified by DAPC.


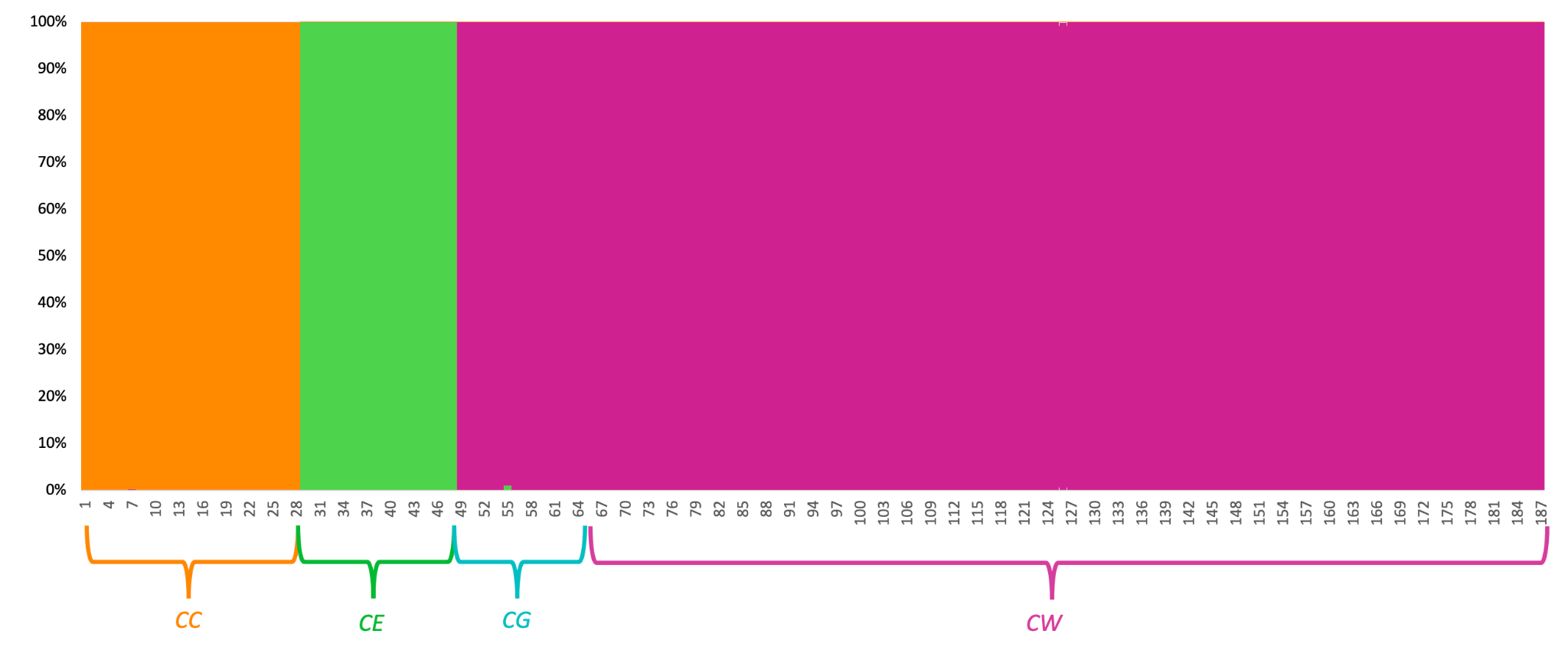


**Supplementary Figure 2.** fastSTRUCTURE plot showing 3 genetic clusters (K=3) colored by genetic identity (Orange = *C. chuckwallensis*, green = *C. echinocarpa* , pink = *C. wolfii* + *C. ganderii*). Each line on the x-axis represents an individual and the proportion of ancestry derived from a certain genetic cluster is represented by the y-axis. **The species abbreviations are as follows :** *C. chuckwallensis* (CC), *C. echinocarpa* (CE)*, C. ganderii* (CG) *and C. wolfii* (CW).


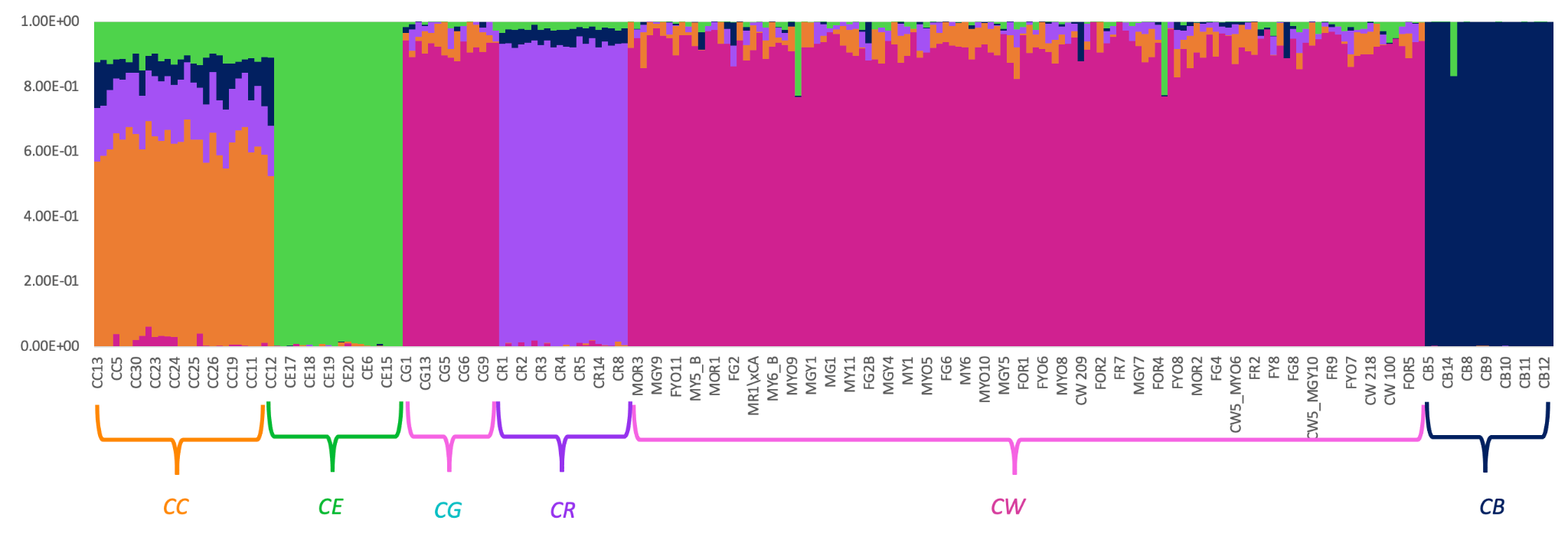


**Supplementary Figure 3.** CONSTRUCT plot based on 10% missing data, showing five genetic clusters (K=5) colored by genetic identity (Orange = *C. chuckwallensis*, green = *C. echinocarpa* , Purple = *C. ramosissima*, pink = *C. wolfii* + *C. ganderii,* Navy *= C. bigelovii*). Each line on the x-axis represents an individual and the proportion of ancestry derived from a certain genetic cluster is represented by the y-axis. **The species abbreviations are as follows:** *C. bigelovii* (CB)*, C. chuckwallensis* (CC), *C. echinocarpa* (CE)*, C. ganderii* (CG) *and C. ramosissima* (CR), *C. wolfii* (CW).
